# Spermatorrhœa

**Published:** 1844-07

**Authors:** T. C. Lewis

**Affiliations:** Wolverhampton


*Spermatorrhœa.*—There exists on this, as on too many other subjects, no small difference of opinion; all observers, however, agree that spermatorrhœa may be produced by onanism, excessive venery, worms, hæmorrhoids, and constipation, which latter, I feel persuaded, is by no means an uncommon cause, more particularly when it is complicated with the former.

Professor Lallemand, of Montpelier, who has paid much attention to the pathology and treatment of this disease, has come to the conclusion that prolonged chastity is, indeed, almost invariably succeeded by spermatorrhœa; and he accounts for this result by asserting that after the age of puberty the seminal fluid cannot with safety be suspended or decreased, for no sooner does it collect than it begets incessant erections and nocturnal or diurnal emissions which cannot be prevented but by sexal congress:—"It is the sole effectual means, both for the *present* and *future.*" His pupil, Dr. Dangerfield, has lately supported the same views.

The late Dr. Ryan of London (Manual of Midwifery, 1831, p. 60), thus expresses himself:—"All healthy persons, at the time of puberty, most certainly feel the passion of physical love. It is a part of their health, and as natural a consequence as *hunger* or *thirst.*" . . . . "It exists *independently* of the will; it may be restrained, but not extinguished. Reproduction seems to be a *common* law of animal and vegetable life, and the disposition to reproduce it in all healthy subjects is most powerful. It is a passion that must be gratified." Again, when speaking of the treatment of impotence from seminal emissions, he maintains that marriage is the natural cure for the complaint; but, nevertheless, although concubinage is immoral, it "is so often acted on, in general, without a medical counsellor, that we must not be too ready to censure the faculty for occasionally proposing it." "Absolute continence," he adds, "from venery, in a healthy subject much disposed to it, induces many dangerous diseases, and even deaths."

M. Cruveilhier, of Paris, on the contrary, does not believe that spermatorrhœa is ever caused by venereal abstinence, nor will he consent to the doctrine that continence is prone to make inroads on the health of individuals. In support of the former, he reminds us of the physiological law "that the less secretion takes place," and in proof of the latter he appeals to his own personal observation among the young priesthood. To these deductions Dr. Bull consents, and contends that Physiological and ethical law can never be hostile to each other.

Dr. Carpenter (Principles of Physiology, 1842) concurs in these opinions, and censures those practitioners who recommend immoral connections. Such advice is "immoral, as well as unscientific." He thinks that the secretion of the testicles is very much under the influence of mind, and it cannot be denied, that his remarks are pertinent. In another part of his volume, however, he writes thus:—"To the use of the sexual organs for the continuance of his race, man is prompted by a *powerful instinctive* desire, which he shares with the inferior animals." . . . "This instinct, when once aroused (even though very obscurely felt), acts upon the mental faculties and moral feelings, and this becomes the source of the tendency to form that kind of attachment towards one of the opposite sex whlch is known as love."

Now, when we meet with such opposing and conflicting statements as the above just enumerated, it appears to me that a more extended and closer investigation is required before we can arrive at just or satisfactory results. The subject is altogether of too much importance to justify us in advancing, as facts, opinions which may be only the fruits of immature observation. At present, then, *lis sub judice est.* Whether continence be productive of spermatorrhœa, or not, in men who have never deviated from the paths of strict rectitude, I cannot undertake to determine; but that it is surely a cause of this disease in those who have at one period of their lives indulged in coition, but have subsequently abstained from it for any considerable time, I most assuredly believe; and that the best, if not the only, cure for malady is a moderate employment of the venereal organs. To this view I have been led from a tolerably fair experience. But I may be wrong; and after all, the whole difficulty in the medical science consists in ascertaining with certainty the precise influence which what we call causes exert on the animal economy. What is proved and received on one day is often disproved and rejected on the morrow. All must acknowledge the force of the objection urged by Cruveilhier. I cannot say that I have ever seen atrophy of the testicles in any healthy and normal individual, and I feel convinced that mental onanism (from which, perhaps, few are free) is alone sufficient to insure the secretion of the seminal fluid and to prevent the degeneration of the virile organs. Eunuchs even, according to Sir A. Cooper, are liable to erections and prostatic emissions.

I have been acquainted with but very few priests, but those whom I have known were healthy-looking persons. It is out of my power to say whether they lead a life of continence or otherwise, but I am aware that some, at least, of our own unmarried clergy gratify their amorous dispositions. I was once consulted by a clergyman, for constipation who practiced masturbation. He had never copulated, but had a great wish to be married; the *res angusta domi* prevented. He defended the practice of onanism, and urged that a man who did not enjoy sexual union was led to it instinctively. John Hunter asserted that masturbation, if confined within proper limits, was not injurious to the system.

But whatever differences of opinion may exist among medical men, in relation to the influence which chastity exerts in the production of spermatorrhœa, one thing is undeniable, namely, that the governments of Europe wink at the practice of illicit connection. In the time of Henry VIII. the stews of London were located in Southwark, and in France stews are publicly recognised at this very day. Moreover, the fact cannot be disguised, that there are few persons who have not given way to this vice, which is called by the historian Gibbon, the most liberal of all, drunkenness being the most illiberal. Although an indulgence in this vice is immoral, and notwithstanding the Saviour has declared that of all sins that of fornication is the most inimical to his religion, yet it does appear that the members of society seem much more willing to defend or excuse it, when confined to single men, than almost any other. Now, although I do not agree with Dr. Bull in believing that continence cannot ever give rise to the inordinate seminal discharges, or be otherwise obnoxious to the general health, but on the contrary, that moderate sexual congress is required to preserve the health, still I cannot either advocate the practice of onanism, or justify fornication. The latter tends greatly to the demoralization of the human species, and is frequently followed by the most wretched ailments. It aims a death-blow to the two great pillars of human society—marriage and parental care. No man either, who endeavours to act up to the precepts inculcated in the New Testament, can consistently or quietly recommend it. Can any believer in Christianity advise his fellow-man (more particularly when he is suffering from a most distressing malady) to spurn the dictates of his Maker to follow the courses of Mammon? But, say others, is he, then, miserably to pine away and perish? I answer, no; let him rather marry, and "eat the bread of carefulness." What, urge a man to disgrace his family and to burthen the state by entering into a matrimonial alliance when he has not the means wherewith to do so? Would any Christian believe that there is a society in being which holds out pecuniary rewards to laborers to remain single till they have attained the age of thirty! Would they have men for a period of eight years go about trying to seduce their own daughters, perhaps, or those of their fellow-men? No; let them restrain their passions. Yes; but that is in the power of very few men to accomplish. If we could, indeed, command our appetites, life might approach truly unto happiness. Erroneous views of life usually lead to unnatural and unfair designs. Knowledge, if not necessary to virtue, is of great assistance to it. We are told by the apostle it is better to marry than burn, and that marriage is honorable to all men; but hear what the shrewd and learned Burton, (himself a divine) says,—"No man shall marry until he be twenty-five, no woman till she be twenty—*nisi aliter dispensatum.* If one die, the other party shall not marry until six months after, and because many families are compelled to live niggardly, exhausted and undone by great dowers, none shall be given at all, or very little, and that by supervisors, rated; they that are foul shall have a greater portion; if fair, none at all, or very little; however, not to exceed such a rate as those supervisors shall think fit. And when once they come to those years *poverty* shall hinder no man from marriage, or any other respect; but all shall be rather *enforced* than hindered, except they be dismembered, or grievously deformed, infirm, or visited with some enormous hereditary disease, in body or in mind, in which cases, upon great pain or mulct, man or woman shall not marry; other order shall be taken from them to their content. If people over-abound, they shall be eased by colonies." (Burton's Anatomy of Melancholy: Democritus to the Reader.)* Of course, we have improved much since Burton's time, and we now have an opportunity of reading the instructive pages of Malthus. "How odious and abominable are those rash vows which bind and enforce men and women to vow virginity, to lead a single life against the laws of nature, opposite to religion, feeling, and humanity! so to starve, to offer violence, to suppress the vigor of youth! and all for base and private respects to maintain their gross superstitions, to enrich themselves by hindering some marriages, that the world be not full of beggars, and their parishes pestered with orphans."

A few words as to *the treatment* and I have done.

The bowels must be kept regular by the use of mild aperients, or (what is better) by means of enemas, which should be thrown up every morning. The genitals and thighs to be sponged with cold water, and the greatest care should be employed to wash away the secretion which accumulates around the corona glandis. The general surface of the body may most advantageously be benefitted by the daily ablution with salt and water. An injection composed of twenty minims of the sedative liquor of opium in water, should be had recourse to every night on going to bed, care being taken to allow it to remain in the urethra for some time. A leech or two may occasionally be applied to the perineum, or under the root of the penis. The solid nitrate of silver I have found an excellent remedy when applied to the orifice of the meatus, but I have not employed it more than once a week. It affords much relief, but it is not permanent. I have never used it as lauded by Lallemand. The patient should sleep with his head high, and on his side: I have generally noticed that seminal emissions occur towards morning, when the person is lying on his back. The disulphate of quinine with sulphuric acid may be exhibited internally, and afterwards iron may gradually be persevered in. Tea and coffee should be avoided, and fresh milk used in their place. There is no objection to the patient's taking mild bitter beer at dinner, but wine must be banished. Great

---

*This quaint, learned, and amusing work should be in the hands of every medical man.

restlessness is a constant attendant on this disease, and opiates have been extolled, but I have never given them a trial. When lettuces are in season one may be taken for supper, about an hour before bed-time. The bed-coverlets should be thrown down towards the foot for some time before the patient retires, and if towards morning the afflicted individual finds that he has had but little repose, he should turn them down again, and walk about the room for a few minutes. By this simple method sleep may often be procured, but under these circumstances he should not rise early. The patient should marry, but he must not indulge in venereal combats more than once or twice a week. Venery is very apt to cause balmy and refreshing repose,—"Finito opere, cupido pacatur, irritatio summovetur, corpus solvitur, et somnus facilius obrepit." All mental onanism must be destroyed, for there can be no doubt that the "lust of the eye" tends greatly to augment the complaint. Until it is perfectly cured, horse-exercise should be strictly prohibited; but walking may be undertaken daily. The attentions should be fixed on some engaging occupation, and the society of indelicate females must be discarded. Frequent changes of scenery are of much service.

Obediently yours,

T. C. Lewis, M.R.C.S., &c.

Wolverhampton, December 19, 1843.

*London Lancet.*

